# TLR9- and CD40-Targeting Vaccination Promotes Human B Cell Maturation and IgG Induction *via* pDC-Dependent Mechanisms in Humanized Mice

**DOI:** 10.3389/fimmu.2021.672143

**Published:** 2021-05-18

**Authors:** Liang Cheng, Guangming Li, Caroline Marnata Pellegry, Fumihiko Yasui, Feng Li, Sandra M. Zurawski, Gerard Zurawski, Yves Levy, Jenny P.-Y. Ting, Lishan Su

**Affiliations:** ^1^ Lineberger Comprehensive Cancer Center, University of North Carolina at Chapel Hill, Chapel Hill, NC, United States; ^2^ Frontier Science Center for Immunology and Metabolism, Medical Research Institute, Wuhan University, Wuhan, China; ^3^ Division of Virology, Pathogenesis and Cancer, Institute of Human Virology, Department of Pharmacology, University of Maryland School of Medicine, Baltimore, MD, United States; ^4^ Department of Microbiology and Cell Biology, Tokyo Metropolitan Institute of Medical Science, Tokyo, Japan; ^5^ Guangzhou Eighth People’s Hospital, Guangzhou Medical University, Guangzhou, China; ^6^ Baylor Institute for Immunology Research, Vaccine Research Institute, INSERM U955, Dallas, TX, United States; ^7^ Assistance Publique-Hôpitaux de Paris, Groupe Henri-Mondor Albert-Chenevier, Service Immunologie Clinique, Créteil, France; ^8^ Vaccine Research Institute, Université Paris-Est Créteil, Faculté de Médecine, INSERM U955, Créteil, France; ^9^ Department of Genetics, University of North Carolina at Chapel Hill, Chapel Hill, NC, United States; ^10^ Department of Microbiology-Immunology, University of North Carolina at Chapel Hill, Chapel Hill, NC, United States

**Keywords:** plasmacytoid dendritic cell, IFN-alpha, immunoglobin class-switch, IgG induction, CD40-targeting vaccination, B cell maturation, CpG-B

## Abstract

Mice reconstituted with a human immune system (humanized mice) provide a robust model to study human immunology, vaccinology, and human infectious diseases. However, the development and function of B cells in humanized mice is impaired. B cells from humanized mice are immature and are impaired in IgM to IgG isotype switch in response to infection or vaccination. In the present study we report that Toll-like receptor 9 (TLR9) agonist CpG-B combined with CD40-targeting vaccination triggered human B cell immunoglobin class-switch from IgM^+^ to IgG^+^ B cells in humanized mice. Human B cells from mice vaccinated with CpG-B as adjuvant were more mature in phenotype and produced significant levels of both total IgG and antigen-specific IgG. We found that CpG-B treatment activated human pDCs (plasmacytoid dendritic cells) *in vivo* to induce interferon-alpha (IFN-α)expression in humanized mice. Pre-depletion of human pDC *in vivo* abrogated the adjuvant effect of CpG-B. Our results indicate that TLR9 and CD40-targeting vaccination triggers human B cell maturation and immunoglobulin class-switch in a pDC-dependent manner in humanized mice. The findings also shed light on induction of human IgG antibodies in humanized mouse models.

## Introduction

Recent development of humanized mice provides robust models to study infection, pathogenesis, and therapy of human viruses (1, 2). We and others have shown before that a functional human immune system was developed in immunodeficient mice after adoptive transfer of human hematopoietic stem cells (HSCs) (3–8). Major human immune subtype such as pDC, mDC, monocyte, T and B cells can be detected in peripheral blood and lymphoid organs 3 months after HSCs transfer (7). Humanized mice can initiate innate immunity and antigen-specific T cell response to vaccine or infection (9–12). However, although B cell are developed in humanized mice, those cells are immature (13). B cells from humanized mice shows CD24^int/hi^CD38^hi^ immature phenotype and express high levels of CD10, another immature B cell marker (14). Moreover, B cells from humanized mice are predominately IgM^+^ with few IgG^+^ B cells (15). The cells are impaired in IgM to IgG isotype switch in response to infection or vaccination (14, 15).

Toll like receptor (TLRs) are expressed by various immune cells such as plasmacytoid dendritic cells (pDCs), monocytes and myeloid dendritic cells (mDCs) and B cells (16, 17). They can sense the microbial components named pathogen-associated molecular patterns (PAMPs) (16, 17). Stimulation of TLR signaling by synthetic or natural TLR ligands (TLR-Ls) results in up-regulation of MHC class II molecules, co-stimulatory molecules, and cytokines in different kind of cells in the immune system, especially innate immune cells (18, 19). These synthetic or natural agonists for TLRs are potential new vaccine adjuvants (20, 21). We and others have shown before that TLR-Ls can efficiently activate human innate immune cells from humanized mice both *in vitro* and *in vivo* (9, 11). Importantly, we proved that CpG-B, R848 and Poly I:C can enhance antigen- specific T cells response to a CD40-targeting HIV vaccine in humanized mice (9). Moreover, we recently reported that therapeutic treatment with a CD40-targeting HIV vaccine plus poly I:C as adjuvant can significantly reduce HIV-1 reservoir in HIV-infected humanized mice (12). These results indicated that humanized mice serve as a relevant model to develop and evaluate novel vaccines and adjuvants to human infectious disease.

As B cells from humanized mice fails to efficiently transition from IgM^+^ to IgG^+^ B cells, it is still questionable to use humanized mouse model to evaluate humoral immune response to vaccines (13). Efforts have been made to improve B cell IgM to IgG class-switch and antigen-specific antibody production in humanized mice in recent years. It was reported that GM-CSF and IL-4 stimulate humoral responses in humanized mice by promoting dendritic cell, T and B cell maturation (22). Transgenes expressing human stem cell factor, granulocyte-macrophage colony stimulating factor and interleukin-3 was reported to improve B cell development in humanized mice (23). The expression of human IL-6 by knocking-in human IL-6 gene to its respective mouse locus also increased class switched memory B cells and serum immunoglobulin G (IgG) after HSCs reconstitution (24). Stimulation of TLR signaling by synthetic or natural TLR ligands (TLR-Ls) results in cytokines induction, as well as up-regulation of MHC class II molecules and co-stimulatory molecules in innate immune cells (9, 11). Synthetic CpG oligodeoxynucleotides (CpG ODNs), which signal through TLR9, are approved by FDA (DYNAVAX) as HBV vaccine adjuvants after human clinical trials. The principal TLR9-expressing cells in humans are plasmacytoid DCs (pDCs) and B cells (25, 26). We speculated that targeting TLR9 would serve as a good stratagem to improve humoral immune response to vaccines in humanized mice. However, knowledge about how CpG ODNs activate pDC and enhance B cell response *in vivo* in humans is still limited.

In the present study we tested whether targeting TLR9 by CpG-B can overcome the deficiency of B cell response in humanized mice. We found that CpG-B combined with CD40-targeting vaccination enhanced B cell maturation, triggered human B cell immunoglobin class-switch from IgM^+^ to IgG^+^ B cells, and induced antigen-specific IgG response. Furthermore, we found that pDCs were essential for the adjuvant activity of CpG-B. Our results indicate that TLR9 and CD40-targeting vaccination triggers human B cell maturation, IgM to IgG immunoglobulin class-switch in a pDC-dependent manner in humanized mice.

## Materials and Methods

### Construction of Humanized Mice

We constructed humanized NRG (NOD-Rag2^-/-^ $\gamma_{C}^{-/-}$) mice by reconstitution with human fetal liver (17 to 22 weeks of gestational age) derived CD34^+^ hematopoietic progenitor cells (Advanced Bioscience Resources, Alameda, CA) as previously reported (27). Briefly, CD34^+^ hematopoietic progenitor cells purified from fetal liver were injected into the liver of newborn (1-3 day) NRG mice. Human immune reconstitution was detected by flow cytometry 12 weeks after transplantation. All animal studies were approved by the University of North Carolina Institutional Animal Care and Use Committee (IACUC).

### Ethics Statement

Human fetal liver was obtained from elective or medically indicated termination of pregnancy through a non-profit intermediary working with outpatient clinics (Advanced Bioscience Resources, Alameda, CA). Informed consent of the maternal donor is obtained in all cases, under regulation governing the clinic. The use of the tissue in the research had no influence on the decision regarding termination of the pregnancy. The project was reviewed by the University’s Office of Human Research Ethics, which has determined that this submission does not constitute human subjects research as defined under federal regulations [45 CFR 46.102 (d or f) and 21 CFR 56.102(c)(e)(l)] and does not require IRB approval. All animal experiments were conducted following NIH guidelines for housing and care of laboratory animals and in accordance with The University of North Carolina at Chapel Hill with protocols approved by the institution’s Institutional Animal Care and Use Committee (IACUC ID: 14-100).

### Vaccination

Recombinant anti-human CD40 antibody fused to 5 HIV-1 long peptide regions (αCD40-HIV5pep) was produced as previously reported (28), except that the flexible linker and HIV peptide sequences were reconfigured and the variable regions CD40 antibody were changed to a human framework. Humanized mice were intramuscularly (half dose) and intraperitoneally (half dose) injected with 10 µg αCD40-HIV5pep or recombinant hemagglutinin protein alone or with 50 µg of CpG-B at week0, week3 and week6. Splenocytes from vaccinated humanized mice were collected 7 days after the third vaccination.

### Detection of Cytokines

Human IFN-α was detected by enzyme-linked immunosorbent assay using the human IFN-α pan ELISA kits purchased from Mabtech. A high sensitivity immunology multiplex assay (Luminex) (Millipore, Billerica, Massachusetts, USA) was used to measure human IL-6 in plasma of humanized mice according to the manufacturer’s instructions.

### Flow Cytometry

Single cell suspensions prepared from peripheral blood, spleen of humanized mice was stained with surface markers and analyzed on a CyAn ADP (Dako). FITC-conjugated anti-human CD40, CD24, PE-conjugated anti-human CD303, CD38, PE/Cy5-conjugated anti-human CD86, IgG, PE/Cy7-conjugated anti-human HLA-DR, PB-conjugated anti-human CD4, IgM, APC-conjugated anti-human CD10 and APC/Cy7-conjugated anti-human CD45 were purchased from Biolegend. Pacific orange-conjugated anti-mouse CD45, PE/Texas red-conjugated anti-human CD19 and LIVE/DEAD Fixable Aqua (LD7) Dead Cell Stain Kit were purchased from Invitrogen. Data were analyzed using Summit4.3 software (Dako).

### Total IgM and IgG Detection

Total IgM and IgG were detected by ELISA kits, purchased from Bethyl Laboratories,int.(Cat. No. E80-104 and Cat. No. E80-100) according to the protocols.

### Detection of Total and Antigen-Specific IgG-Secreting Cells by ELISpot

IgG-secreting cells were detected by using ELISpot^PLUS^ for Human IgG kits (Product Code: 3850-2HW-Plus) according to the protocols. In brief, 96-well ELISpot plates were coated with an IgG capture antibody (for total IgG detection) or antigen (αCD40-HIV5pep, for specific IgG detection) in PBS overnight at 4°C. Then the plate was blocked with 200 μl/well of medium containing 10% FBS for at least 30 minutes at room temperature. The pre-activated splenocytes (splenocyte from humanized mice cultured ex vivo in the present of R848(1μg/ml) and IL-2 (10 u/ml) for 48hours) were then added to the ELISpot plate and incubated in a 37°C incubator with 5% CO_2_ for 16-24 hours. Plates were washed 5 times with PBS and incubated with IgG detection mAbs for 2 hours at room temperature. Then, 1:1,000 dilution of streptavidin-HRP was added and incubated for 1 hour at room temperature. Followed by washing 5 times, the plates were developed with TMB substrate solution until distinct spots emerge. The spots were inspected and counted by using an ELISpot reader.

### Antigen-Specific IgG Detection in Serum

96-well ELISA plates were coated with antigen (αCD40-HIV5pep, 10 µg/ml or HA protein, 10µg/ml) in PBS overnight at 4°C. Then the plate was blocked with 200 μl/well of medium containing 10% FBS for at least 30 minutes at room temperature. Then 50 μl of serum from vaccinated mice was added into the plate and incubated for 2 hours. Plates were washed 5 times with PBS and incubated with IgG detection mAbs for 2 hours at room temperature. Then, 1:1,000 dilution of streptavidin-HRP was added and incubated for 1 hour at room temperature. Followed by washing 5 times, the plates were developed with TMB substrate solution. The reaction was stopped with ELISA Stop Solution (Cat. No. E115), and the plate was read at 450 nm.

### pDC Depletion *In Vivo*

A mAB specific to BDCA2, clone 15B, was used to deplete pDCs in humanized mice through i.p. injection (4 mg/kg). In brief, 15B was applied to mice at 3 and 1 day before each vaccination. At day0, the mice were either treated with CpG-B or received vaccination treatment.

### Statistical Analysis

In all experiments, significance levels of data were determined by using Prism5 (GraphPad Software). Experiments were analyzed by two-tailed Student’s t-test, one-way analysis of variance (ANOVA) and Tukey’s multiple comparisons test, or Spearman rank correlation test as indicated in figure legends. A p value less than 0.05 was considered significant. The number of animals was specified in each figure legend.

## Results

### TLR9 and CD40-Targeting Vaccination Promotes IgG Induction in Humanized Mice

**Figure 1:**
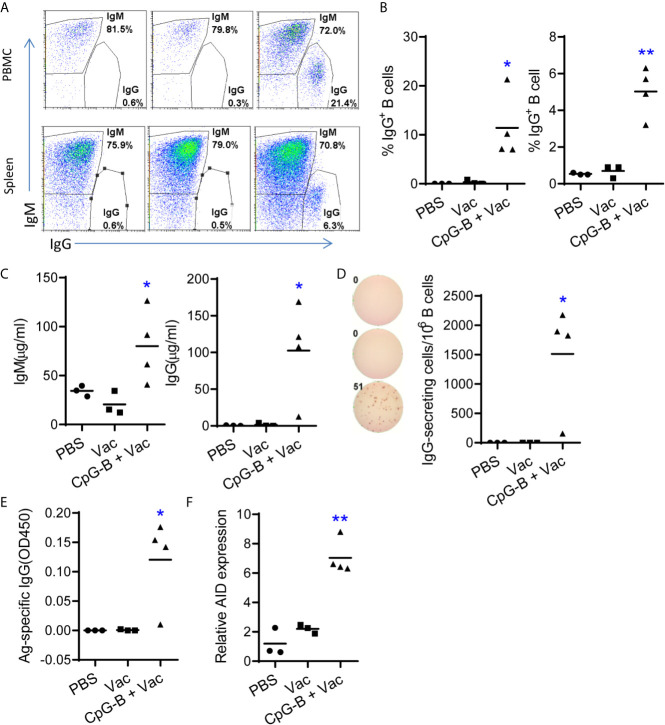
TLR9 agonist CpG-B promotes IgG responses in humanized mice vaccinated with CD40-targeting vaccine. Humanized mice were treated with PBS control (n=3) or vaccinated with αCD40-HIV5pep (n=3) alone or vaccinated with αCD40-HIV5pep plus CpG-B (n=4) at week0, week3 and week6. At week 7, mice were sacrificed. **(A, B)** The expression of IgM and IgG on B cells from PBMCs and spleens was detected by FACS. **(C)** The total level of IgM and IgG in the plasma was detected by ELISA. **(D)** Splenocyte from humanized mice were cultured ex vivo in the present of R848(1μg/ml) and IL-2(10 u/ml) for 48hours, the cells were used for total IgG detection by ELISpot. **(E)** Antigen specific IgG level in the plasma was detected by ELISA. **(F)** The expression of activation-induced cytidine deaminase (AID) in spleen cells was detected by RT-PCR. Each dot represents one individual mouse, bars indicate mean. *P < 0.05, **P < 0.01, by unpaired, two-tailed Student’s t-test comparing the two vaccinated groups.

Human B cell development and function are compromised in humanized mice, and B cells are impaired to undergo immunoglobin class switch from IgM to IgG. We tested whether targeting TLR-9 by CpG-B combined with a CD40-targeting vaccine can enhance B cell immunity in humanized mice. Humanized mice were immunized with αCD40-HIV5pep protein with or without CpG-B. B cell responses were evaluated one week after the boost vaccination. As reported (15), we found that B cells from PBMCs and spleen of PBS-treated humanized mice did not express surface IgG (**Figures 1A, B**). Vaccination with αCD40-HIV5pep protein alone failed to induce IgG expression on B cells (**Figures 1A, B**). Impressively, we found that, in humanized mice vaccinated with CpG-B together with αCD40-HIV5pep protein, around 12% (7.0% to 21.3%) of B cells from PBMCs and 5% (3.2% to 6.3%) of B cells from the spleens of humanized mice were IgG^+^ B cells (**Figures 1A, B**). CpG-B together with αCD40-HIV5pep vaccination also increased total human IgM level by-2 fold (**Figure 1C**). Human IgG, which is rarely detectable in humanized mice, reached 100 μg/ml in 75% (3/4) humanized mice after CpG-B together with CD40-targeting vaccination (**Figure 1C**). We also detected by ELISpot assay around 1,500 cells per million B cells from spleens of 75% (3/4) humanized mice received CpG-B plus CD40-targeting vaccination that were producing IgG, while no IgG-producing cells were detected in control groups (**Figure 1D**).

We next tested whether antigen-specific IgG was induced by CD40-targeting vaccination together with CpG-B. We found that 75% (3/4) mice from CpG-B/CD40-targeting vaccine group produced specific IgG, while no antigen specific IgG was detectable in control groups (**Figure 1E**). Expression of activation-induced cytidine deaminase (AID) by germinal center (GC) B cells is important for class switch and recombination (29). We found that AID expression was significantly increased (> 3-fold) in splenocytes from CpG-B plus CD40-targeting vaccination mice than cells from control groups (**Figure 1F**).

Together, our data indicate that TLR9 agonist CpG-B with a CD40-targeting vaccine induces immunoglobulin class-switch and enhances Ag-specific IgG responses in 75% (3/4) humanized mice tested.

### CpG-B Promotes Maturation and Activation of B Cells in Humanized Mice

**Figure 2:**
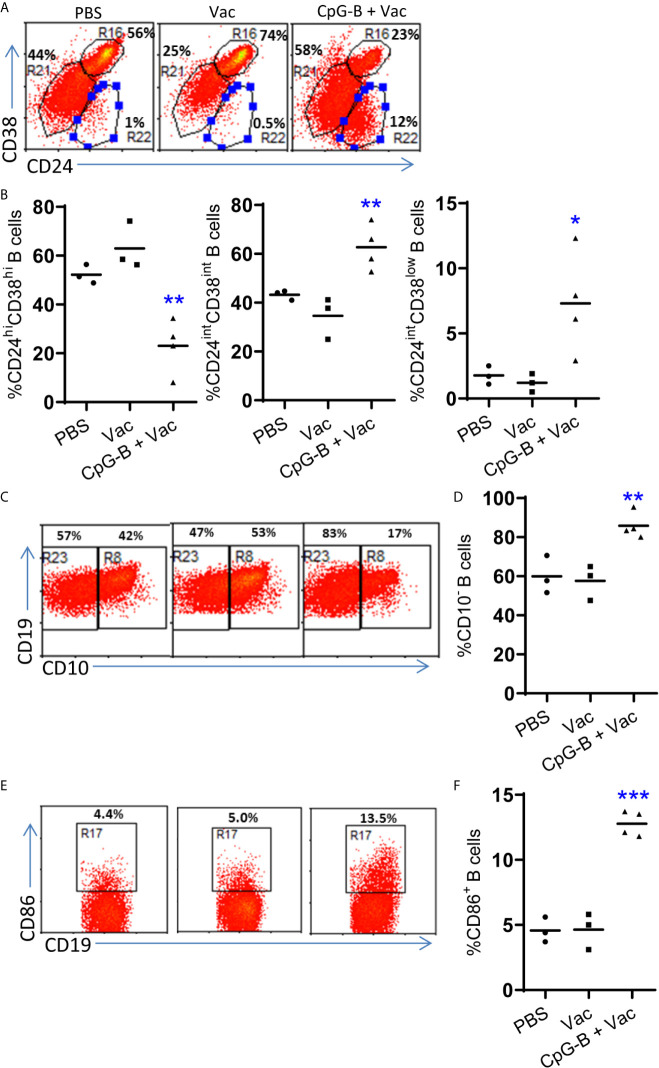
CpG-B promotes maturation and activation of B cells in humanized mice. Humanized mice were vaccinated as in **Figure 1**. The phenotype of human B cells (hCD45^+^CD19^+^) from spleens of mice was detected by FACS. Representative dot plot **(A, C, E)** and Summarized data **(B, D, F)** showing the expression of CD38and CD24, CD10 and CD86 on human B cells. Each dot represents one individual mouse with n=3 in PBS group, n=4 in Vac group and n=4 in CpG-B plus Vac group. Bars indicate mean. *P < 0.05, **P < 0.01, ***P < 0.001, by unpaired, two-tailed Student’s t-test comparing the two vaccinated groups.

We next detected the phenotype of human B cells after vaccination. As reported (14), we found that most B cells from control mice were CD24^high^CD38^high^ which indicated immature phenotype (**Figures 2A, B**). CD40-targeting vaccination alone did not change the percentage of CD38^high^CD24^high^ immature B cells. CpG-B together with CD40-targeting vaccination decreased the percentage of CD38^high^CD24^high^ B cells and increased the percentage of mature B cells with CD38^int^CD24^int^ or CD38^low^CD24^int^ phenotype (**Figures 2A, B**). We also find that CpG-B together with CD40-targeting vaccination increased the percentage of CD10^-^ B cells (**Figures 2C, D**). In addition, CpG-B together with CD40-targeting vaccination induced CD86^+^ activated B cells (**Figures 2E, F**). These data indicated that CpG-B plus CD40-targeting vaccination induce B cells maturation and activation.

### CpG-B Induces IFN- α Production in pDC Dependent Manner in Humanized Mice

**Figure 3:**
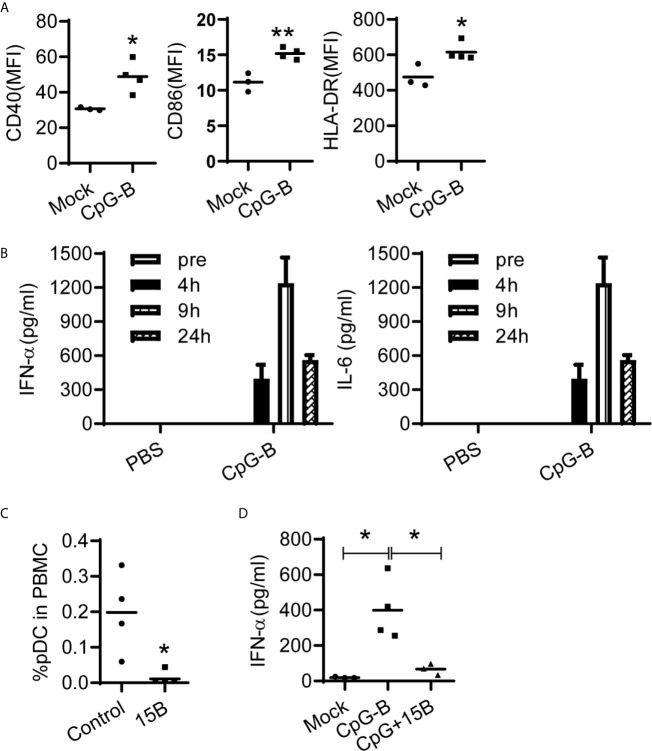
CpG-induced IFN-a production *in vivo* is dependent on human pDCs in humanized mice. **(A, B)** Humanized mice were treated with PBS (n=3) or CpG-B (50ug/mouse, i.p., n=4). **(A)** IFN-α and IL-6 level in plasma at indicated timepoint post treatment were detected by ELISA. **(B)** The expression of CD40, CD86 and HLA-DR on pDC (CD4^+^CD303^+^) from spleens at 24 hours post-treatment was detected by FACS. **(C)** Humanized mice were pretreated with either isotype control (n=3) or pDC depletion monoclonal antibody (clone 15B, 200 μg/mouse, i.p., n=4) at day -3 and -1. The percentage of pDC in human CD45+ cells from blood were detected by FACS. **(D)** Humanized mice were pretreated with either isotype control (n=4) or pDC depletion monocolonal antibody (clone 15B, 200 μg/mouse, i.p., n=3) at day -3 and -1. At day0, mice were treated PBS (Mock, n=4) with CpG-B (n=4). IFN-a level in plasma was detected 24 hours after CpG-B treatment. Each dot represents one individual mouse, bars indicate mean. *P < 0.05, **P < 0.01, by unpaired, two-tailed Student’s t-test **(A, C)** or by one-way analysis of variance (ANOVA) and Tukey’s multiple comparisons test **(D)**.

Dendritic cells are key to initiate and control immune responses. Plasmacytoid dendritic cells (pDC) represent a unique dendritic cell subtype and are specialized to produce large amount of IFN-α upon viral infection (30). It was reported that pDCs, through IFN-α and IL-6, are critical for the induction of IgG from human blood mononuclear cells in response to influenza virus (31). We found that CpG-B treatment activated human pDCs (**Figure 3A**) and induced IFN-α and IL-6 production in humanized mice (**Figure 3B**). TLR9, the receptor for CpG-B, is preferentially expressed by pDC and B cells of human immune system. To investigate whether pDC contributed to IFN-α production after CpG-B treatment, we used a monoclonal antibody which can efficiently and specifically deplete pDC *in vivo* in humanized mice (32) (**Figure 3C**). We found that pre-depletion of pDC abrogated IFN-α production after CpG-B treatment (**Figure 3D**). The results indicate that CpG-B treatment in humanized mice induces IFN-α expression thought a pDC dependent manner.

### TLR9 and CD40-Targeting Vaccination Depends on pDC to Promote Human B Cell Maturation and IgG Induction in Humanized Mice

**Figure 4:**
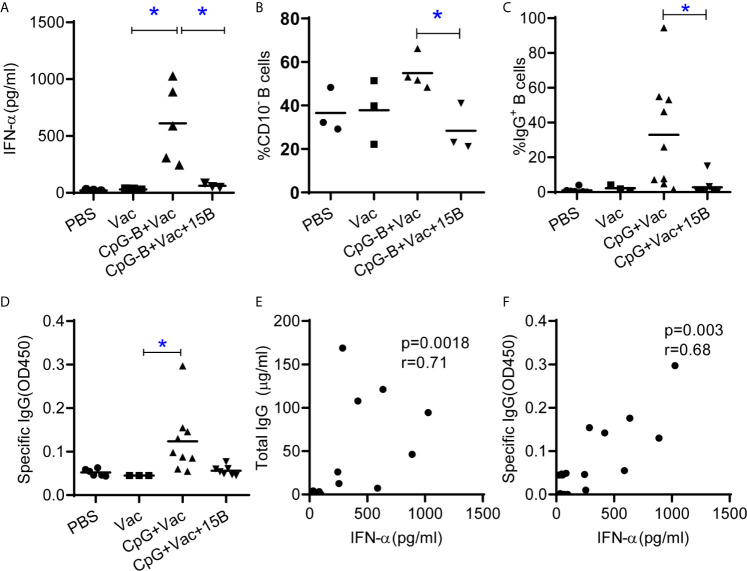
TLR9 and CD40-targeting vaccination depends on pDC to promote human B cell maturation and IgG induction in humanized mice. Humanized mice were vaccinated as in **Figure1** except one group of the mice were treated with pDC depletion Ab before vaccination **(A)** IFN-α in the plasma 24 hours after vaccination. **(B)** Expression of CD10 on B cells from spleen at termination. **(C)** Total IgG level in serum was detected by ELISA. **(D)** Antigen specific IgG level in the plasma was detected by ELISA. Each dot represents one individual mouse, bars indicate mean. Shown are representative data (PBS, n=3; Vac, n=3; CpG-B+Vac, n=4; CpG+Vac+15B, n=3, for A and B) or combined data (PBS, n=6; Vac, n=3; CpG-B+Vac, n=9; CpG+Vac+15B, n=7, for C and D) of two independent experiments with mean values. *P < 0.05, by one-way analysis of variance (ANOVA) and Tukey’s multiple comparisons test. **(E, F)** Correlation analysis between the IFN-α levels in plasma and total IgG **(E)** and specific IgG **(F)** levels in plasma (Spearman rank correlation test). r, correlation coefficient.

We next investigated whether CpG-B and CD40-targeting vaccination induced B cell maturation and IgG production *in vivo* dependent on pDC. We vaccinated humanized mice with CpG-B and CD40-targeting vaccine in the presence or absence of pDC and then detected B cell phenotype and antibody response. As expected, pre-depletion of pDC by monoclonal antibody abrogated CpG-B induced IFN-α production (**Figure 4A**). Interestingly, CpG-B and CD40-targeting vaccine induced B cell maturation was also abrogated in the absence of pDC (**Figure 4B**). In the absence of pDC, CpG-B and CD40-targeting vaccination also failed to induce total IgG (**Figure 4C**) and antigen-specific IgG (**Figure 4D**). Furthermore, we found that the IFN-α level in plasma of mice correlated with total and specific IgG production in vaccinated hu-mice (**Figures 4E, F**).

### CD40-Targeting Is Important for the Vaccine to Induce Class-Switch and IgG Production

**Figure 5:**
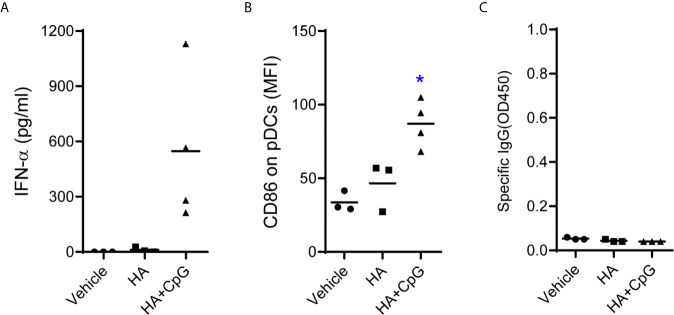
CD40-targeting vaccination is required to induce IgG-response in humanized mice. Humanized mice were treated with PBS control (Vehicle, n=3) or vaccinated with recombinant hemagglutinin protein (HA, n=3, 10μg/mouse) alone or vaccinated with HA plus CpG-B (n=4) at week0, week3 and week6. **(A)** IFN-a levels in plasma in plasma at 24 hours post first vaccination was detected by ELISA. **(B)** The expression of CD86 on pDC (CD4^+^CD303^+^) from PBMC at 24 hours post-treatment was detected by FACS. **(C)** Antigen specific IgG level in the plasma was detected by ELISA. Each dot represents one individual mouse, bars indicate mean. *P < 0.05 by unpaired, two-tailed Student’s t-test comparing the two vaccinated groups.

CD40 is a co-stimulatory receptor expressed by a range of APCs, including DCs (33). Thus, targeting CD40 offers the potential advantage of inducing DC maturation and delivery of antigen to CD40 induced antigen-specific humoral and cellular immune response (34, 35). We next determined whether CD40-targeting is important to induce class-switch and IgG production in humanized mice. Humanized mice were immunized with recombinant hemagglutinin protein (HA) with or without CpG-B. HA protein alone failed to induce specific IgG (**Figure 5**). HA with CpG-B as adjuvant, although induce IFN-α production and CD86 expression on pDC, also did not induce specific IgG response to HA protein (**Figure 5**). These results indicate that CD40-targeting is important for the vaccine to induce class-switch and IgG production.

Taken together, we conclude that TLR9- and CD40-targeting vaccination promotes human B cell maturation and IgG response *via* pDCs dependent mechanisms in humanized mice. The study also provides insights for using humanized mouse models for vaccine development and induction of human IgG antibodies in humanized mice.

## Discussion

Humoral immunity is compromised in humanized mice (13). In the present study we reported that CpG-B adjuvant combined with a CD40-targeting vaccination enhanced antigen-specific IgG response. Furthermore, we proved that pre-depletion of human pDC *in vivo* abrogated the adjuvant effect of CpG-B. Our results indicated that CpG-B and CD40-targeting vaccination promoted B cell maturation, triggered human B cell immunoglobulin class-switch and induced IgG production in a pDC-dependent manner in humanized mice. The findings also shed light on induction of human IgG antibodies in humanized mouse models.

Mice reconstituted with human immune system provides a useful model to study human immunology and vaccinology. Although B cells are developed in humanized mice reconstituted with human HSCs, the cells are immature (13–15, 22–24). The circulating antibody levels, especially IgG levels, are significantly lower compared to adult humans (13). Moreover, the generation of antigen-specific IgG responses in humanized mice, are weak, limiting their application in testing candidate vaccines (13). It was reported that the immunoglobulin gene repertoires of human B cells from humanized mice are similar to those of B cells from human peripheral blood, suggesting that B cells from humanized mice have the genetic potential to produce antibody responses with broad isotype and high affinity (36). Studies have suggested that an absence of human cytokines and the disorganized secondary lymphoid structures might contribute to the defects in B cells (37–39). Signaling TLR results in large amount of cytokines induction in humanized mice. It was reported that pDC-derived IFN-α and IL-6 were critical for the induction of IgG from human blood mononuclear cells in response to influenza virus (31). pDC preferentially express TLR9 and TLR7 (25). We have previously shown that stimulation of pDC by TLR-9 agonist CpG-B induces IFN-α as well as IL-6 production *in vivo* in humanized mice (9). In the present study, we found that CpG-B adjuvant combined with CD40-targeting vaccination induced IgG^+^ B cells in spleen and PBMCs of humanized mice and induced antigen-specific IgG in serum. This indicated that CpG-B and CD40-targeting vaccination triggers human B cell immunoglobulin class-switch in humanized mice. We also observed that B cells from mice receiving CpG-B plus CD40-targeting vaccination were more mature in phenotype and express a higher level of the activation marker CD86. The results together suggest that with proper adjuvant, human B cells in this model can produce specific IgG to a vaccine treatment. We also performed CpG-B plus non-CD40 targeting recombinant protein and showed that it did not induce significant IgG response. Thus CD40-targeting, as well as TLR9 activation, was also important for the vaccine to induce class-switch and IgG production. This could be due to CD40 activation of targeted APCs or prolonged antigen-presentation as was observed for T cell epitopes (40).

To prove whether pDCs are important for the IgG induction *in vivo*, we used a pDC-specific antibody to deplete pDC before vaccination. Our results showed that depletion of human pDC *in vivo* abrogated the B cell maturation and IgG production in response to CpG-B/CD40-targeting vaccination. The results suggest that human pDCs are essential to mediate the adjuvant effect of CpG-B *in vivo*. This is consistent with the report that pDC-derived IFN-α and IL-6 were critical for the induction of IgG from human blood mononuclear cells in response to influenza virus (31). We found that pDCs were required for CpG-B treatment in humanized mice to induce IFN-α and IL-6 production. We speculate that IFN-α and IL-6 from pDC enhanced B cell maturation and IgG-class switch in response to CpG-B plus CD40-targeting vaccination. It is also important to state that there were shortcomings in our study. The antigen we used in this study was not HIV envelop protein so that we were not able to evaluate the neutralization activity of antibody induced by our vaccination stratagem. In another recent study, we proved that TLR-9 agonist plus CD40-targeting HIV envelop vaccination induced HIV-1 envelop-specific IgG with a diversified immunoglobulin repertoire and circulating Env-specific IgG-switched memory human B cells that exhibit clear signs of antigen-driven antibody affinity maturation (41).

In summary, we report that CpG-B as an adjuvant promoted human IgG induction through pDC-dependent mechanisms. The proof-of-concept study sheds light on specific induction of human IgG antibodies in humanized mice.

## Data Availability Statement

The original contributions presented in the study are included in the article/supplementary material. Further inquiries can be directed to the corresponding authors.

## Ethics Statement

The animal study was reviewed and approved by University of North Carolina Institutional Animal Care and Use Committee.

## Author Contributions

LC and LS conceived the study and designed the experiments. GL, FL, FY, and CP performed the experiments. LC performed the analyses. LC, JT, and LS interpreted the data, wrote the manuscript, and supervised the study. GZ and SZ provided key reagents. YL helped conceive the overall program for studying anti-CD40 targeting in humanized mice. All authors contributed to the article and approved the submitted version.

## Funding

This study was supported in part by NIH grants R01AI136990 (to LS), AI141333 (to JT), AI109784 (to JT and LS), and the Vaccine Research Institute *via* the ANR-10-LABX-77 grant funded part of this work.

## Conflict of Interest

GZ, SZ, and YL are named inventors on patent applications relevant to αCD40-HIV5pep held by INSERM Transfert.

The remaining authors declare that the research was conducted in the absence of any commercial or financial relationships that could be construed as a potential conflict of interest.
